# Retraction: Up-regulation of long non-coding RNA SNHG20 promotes ovarian cancer progression via Wnt/β-catenin signaling

**DOI:** 10.1042/BSR20170681_RET

**Published:** 2025-06-23

This article is being retracted from *Bioscience Reports* at the request of the Editor-in-Chief and the Editorial Board. This follows the receipt of a notification from a reader, alerting the Editorial Board to two duplications in the western blot data of Figure 4B and 4E. Specifically, the Figure 4B SKOV3/GAPDH bands appear the same as the Figure 4E GSK-3B/OVCA429 bands, and the Figure 4E p-GSK-3B/siNC band appears the same as the GSK-3B/siSNHG20 band. The Materials and Methods section of the paper also describes lung cancer samples, rather than ovarian cancer, and the phrases are consistent with articles from other author groups: https://doi.org/10.1080/15384101.2017.1301334 and https://doi.org/10.1016/j.bbrc.2017.03.042

The authors were contacted regarding the concerns raised and provided western blot data, however not in the unedited, uncropped raw format requested by the journal. They also provided an explanation for the reference to lung cancer, but not how it corresponded with the other articles. The data and the explanation provided have not alleviated the journal’s concerns. The authors were contacted regarding the retraction but have not responded; given the extent of the issues raised, the Editorial Board stands by the decision to retract the article.

